# Out of the labs and into the streets: Effects of climate protests by environmental scientists

**DOI:** 10.1098/rsos.241001

**Published:** 2025-04-23

**Authors:** Fabian Dablander, Maien S. M. Sachisthal, Adam R. Aron

**Affiliations:** ^1^ Institute for Biodiversity and Ecosystem Dynamics, University of Amsterdam, The Netherlands; ^2^ Institute for Advanced Study, University of Amsterdam, The Netherlands; ^3^ Department of Social Psychology, University of Amsterdam, The Netherlands; ^4^ Department of Psychology, University of California San Diego, La Jolla, CA, USA

**Keywords:** climate change, climate activism, scientist engagement

## Abstract

There have been increasing calls for scientists to ‘get out of the labs and into the streets’ and become more involved in climate change advocacy and protest, including civil disobedience. A growing number of scientists are heeding these calls, but the potential impact of such engagement on the public and the credibility of science remains critically understudied. In this registered report, we used a vignette approach to examine the potential effects of scientists’ engagement in two types of protest in a large representative sample (in terms of age and gender; *n* = 2856) of people in the United States, taking into account political affiliation. Contrary to our predictions, we found that an environmental scientist’s endorsement of or involvement in a protest did not reduce public support for oil and gas drilling, increase support for activists or alter perceptions of protest radicalness. As predicted, we found that scientists’ participation in protests did not reduce the public’s reported level of credibility of the participating scientists or of environmental scientists more broadly. These findings suggest that scientists can engage in public protest without compromising their credibility, but that such actions alone may have less impact than one would like to believe.

## 1. Introduction

Despite urgent warnings about the consequences of continuing on our current trajectory, global action on climate change remains inadequate [1,2]. There is a growing recognition that insufficient mitigation and adaptation to climate change is substantially owing to resistance from actors who benefit from the status quo [3–6]. It has been argued that overcoming this resistance and compelling governments and corporations to take decisive action against climate change requires bottom-up pressure from large parts of society [7–9].

There have been increasing calls that scientists and academics, one of the most trusted groups in society [10,11], could help build this pressure by engaging in more advocacy and protest, including civil disobedience [12–16] — ‘moving out of the labs and into the streets’ [17,18]. A recent global study on climate change engagement found that more scientists agree than disagree that scientists should engage more in advocacy and protest, and almost half of all participants said they are willing to engage in civil disobedience [19]. The study also found that around a quarter of respondents, in particular those working on climate change, reported engaging in protest [20]. Civil disobedience by scientists is increasingly visible too: climate action groups composed of scientists such as Scientist Rebellion (http://scientistrebellion.org/) are operating in over 30 countries, engaging in civil disobedience actions from blocking roads, coal mines and private jets to occupying the offices of high-polluting industries and government representatives [18].

While some argue that scientists engaging in protest and civil disobedience may support the wider climate movement by communicating the urgency of the problem (e.g. [12]), others argue that the political engagement of scientists may harm credibility and should be avoided (e.g. [21,22]). Yet to date, little empirical research has focused on the effects of scientists engaging in legal protests, such as marches, and civil disobedience on the public’s perception of climate change and science credibility; most empirical research has instead been focused on advocacy (e.g. [23–28]) or on the effect of the extremeness of protest actions on activist support [29,30].

Indeed, to our knowledge, there is only one study that investigated the effects of scientists engaging in civil disobedience. Using a vignette study where participants read about the effects of climate change-driven ‘wind stilling’ (i.e. reductions in global average surface wind speed), Friedman [31] found a statistically significant increase in perceptions of risk to humanity when the media article described an environmental scientist who engaged in civil disobedience compared to one that simply urges individuals to take action, but no statistically significant difference in the credibility of the scientist. However, this study’s generalizability is limited owing to the use of a convenience sample of undergraduates. Moreover, while the study did not find differences of effects based on political affiliation, participants who identified as Republican in the United States (US) were under-represented, making inferences about political affiliation inconclusive.

Here, we test the effects of climate protest (legal march vs civil disobedience) and scientist engagement (control vs endorse vs engage) on a set of more comprehensive outcomes, namely policy support, activist support, perceived radicalness of tactics, science credibility (source and general) and donation behaviour. We now discuss these briefly in turn. One key aim of climate advocacy and activism is to strengthen policies designed to address climate change. It is therefore critical to assess whether scientists engaging in protest and civil disobedience can increase the public’s support for the policy in question. We also assess whether scientists’ engagement can increase the public’s support of activists, which is generally low (e.g. [32]), and reduce the perceived radicalness of the protest. Next, we assess effects on the credibility (sometimes also referred to as trustworthiness, cf [25]) of environmental sciences in general and the individual engaged scientist in particular. Finally, we measure real behaviour by recording the amount of prize money the participants donate. More specifically, participants are told that they have the chance to win an additional $100 in payment as prize money and can then choose to donate any amount they wish to a not-for-profit organization working on climate change (e.g. Rainforest Action Network, Friends of the Earth, Climate Emergency Fund) of their choosing. We use a representative sample of the US with respect to age and sex and take into account political affiliation, distinguishing between Democrats, Independents and Republicans, which is important given that climate change is heavily polarized [29,33].

We draw on different strands of research in formulating our hypotheses. First, concerning protest form (legal march vs civil disobedience); while Friedman [31] found higher perceived risk perceptions when a scientist engaged in civil disobedience, a recent panel data study assessing the effect of climate protests by citizens in Germany found that the increase in climate concern did not differ significantly across different types of protests [34]. We therefore expect no difference for the effect of protest form on policy support by the public (H1). Concerning activist support and perceived radicalness of the protest, we expect more support for activists (H2) and less perceived radicalness (H3) in the legal march compared with the civil disobedience condition. This would be in line with earlier research showing less support of activists and higher perceived radicalness for protests using more extreme forms of protest [30,35]. We have no expectations concerning the effect of the protest form on donation behaviour. We do not test the effect of the protest form on science credibility.

Second, for different types of scientist engagement (control vs endorse vs engage), we expect the highest levels of policy (H4) and activist support (H5) for the scientist engaging condition, followed by the endorse condition and then by the control condition. We base these hypotheses partially on Friedman [31], who found that scientist engagement in civil disobedience led to higher risk perceptions concerning effects of climate change, and Capstick *et al*. [12], who argue that scientists engaging in civil disobedience can increase support for the wider climate movement. However, we do not make a distinction between the protest form, and we also include the hypothesis that the endorse condition will elevate policy and activist support compared with the control condition. This is not based on prior research, but makes intuitive sense given that scientists’ engagement can be understood as expressing the strongest support, with expressing endorsement only verbally being in between such engagement and no engagement at all. Given that scientists are one of the most trusted groups in society [10,11], we expect that legal marches (H6) and civil disobedience (H7) are perceived as the least radical for the scientist engaging condition, followed by the endorsing condition and then followed by the control condition. We do not expect engagement type to influence source nor general science credibility (H8, H9), which would be consistent with the findings of Friedman [31]. We have no expectation concerning the effects of scientist engagement type on donation behaviour.

Third, for political affiliation (Democrat, Republican or Independent), while one might naturally expect particular main effects, for example that Republicans will indicate lower levels of policy support [36,37] and activist support [29,30], political affiliation is not of central interest to this study. Indeed, prior research is inconclusive regarding potential interaction effects between political affiliation and protest form and engagement type. While Motta [38] found a polarizing effect of the ‘March for Science’ on attitudes towards scientists based on political affiliation, vignette studies both on perceived radicalness of protests on activist support [30] and on scientists’ credibility following (advocacy) engagement [26,31] did not find that effects differed based on political affiliation. Some studies did find differences based on political affiliation: while activist support increased for both Democrats and Independents after being presented with descriptions of both a legal march and a civil disobedience action, protest form did not influence Republicans [29]. Political affiliation was also found to influence messaging effects of scientists promoting climate policy, both on perceived urgency and science credibility [27], with backfire effects of some messages for both Republicans and Independents. Given the inconclusive results, we formulate no specific expectations on interaction effects. We do, however, assess potential main and interaction effects of political affiliation as well as protest form and type of scientists engagement using exploratory analyses.

## 2. Methods

### 2.1. Design

We use a 2 (protest form) × 3 (type of scientist engagement) × 3 (political affiliation) between-subject design. The type of scientist engagement includes a control condition in which only ‘normal’ citizens engage in the protest form; a condition in which citizens engage in the protest and a scientist (Dr Alex Fraser, Environmental scientist at Rochester University) endorses the action but does not engage in the protest themselves; and a condition in which the scientist engages in the protest themselves. The protest form is either a legal climate change related protest or a civil disobedience action for which protesters are arrested. The political affiliation is either Democrat, Republican or Independent. The study design was approved by the local ethics committee of the Faculty of Social and Behavioural Sciences of the University of Amsterdam (protocol number FMG-8675).

### 2.2. Analysis plan

We used confirmatory hypothesis testing to assess evidence for our pre-specified hypotheses (see the electronic supplementary material, table S1), and exploratory analyses to assess whether any interactions, including with political affiliation, exist. Using linear models to analyse ordinal data can lead to increased false alarms, lower statistical power, distorted estimates of effect sizes and even inversions of differences between groups [39,40]. We therefore used ordinal regression to analyse dependent variables that are five-point Likert items. We used Bayes factors to quantify the evidence in our data for or against our hypotheses in a continuous manner (cf [41,42]). In particular, we used a Bayesian ordinal regression model with sum coding in the R package *brms* [39,43]. In the ordinal two group case, this means


$$\mu_{1}=\;-\delta \;/\;2$$

$$\mu_{2}=\;+\delta \;/\;2,$$

where $\mu_{1}$ and $\mu_{2}$ are the latent group means and δ is the difference between them. We used a cumulative ordinal model which assumes that the latent variables follow a standard Gaussian distribution. In addition to the effect size δ , the model has four threshold or intercept parameters, which slice up the latent variable to yield the outcome Y∈[1,2,3,4,5] and which are constant across groups. We used zero-centred *t*-distributions with 3 degrees of freedom and 2.5 as scale parameter for these threshold parameters, and a Cauchy distribution with a scale parameter of 0.30 for δ [44]. The prior for the threshold parameters does not matter for testing, and hence we used a relatively wide weakly informative prior [45]. The prior choice for the effect size was informed by Friedman [31], who found Cohen’s *d* effect sizes of around 0.35. Our Cauchy prior implied that differences between the latent groups are centred at zero and that 50% of the probability mass is within the [−0.30, 0.30] range. We used sensitivity analyses to assess how robust our conclusions are to different prior widths, ranging from 0.10 to 0.50 for the effect size (see the electronic supplementary material). We considered a Bayes factor larger than 10 as sufficient evidence to support a hypothesis, albeit noting that the Bayes factor is a continuous measure of evidence and should be understood as such [41]. We applied this threshold only when contrasting the constrained model (i.e. a model where the latent means are ordered in a particular way) or the full model with the null model, because the Bayes factor is bounded for comparisons between the constrained model and the full model: for two groups, the maximum Bayes factor in favour of the constrained model is two, for three groups it is six (cf [46]). For assessing evidence in favour of a directed hypothesis, we first contrast the constrained model and the null model. If this Bayes factor was sufficiently large (i.e. larger than 10), and the Bayes factor of the constrained model against the full model is larger than one, we concluded that our directed hypothesis is substantiated. For hypotheses where we expected no effect or did not have a predicted direction (e.g. donation behaviour), we compared only the full model and the null model. We refer to Bayes factors in favour of a hypothesis between 1 and 3 as indicating weak, between 3 and 10 as moderate, between 10 and 30 as strong and above 30 as very strong evidence (cf [47,48]).

In the ordinal three-group case, for which we used the same prior specification as in the two-group case, using sum coding as above we had:


$$\mu_{1}=\;\delta_{1},$$

$$\mu_{2}=\;\delta_{2}$$

$$\mu_{3}=\;-\left(\delta_{1}+\delta_{2}\right).$$

For hypotheses that include only two groups, we used either directed (H2, H3, H6, H7) or undirected ordinal tests (H1, H8). For hypotheses that include three groups, we also used either directed (H4, H5) or undirected (H9) tests. For the directed tests for two groups, we compared models that assume no difference (null model), assume any difference (full model) and assume a difference in the hypothesized direction (constrained model). For the directed tests for three groups, we did the same, specifying $\mu_{1}>\mu_{2}>\mu_{3}$ for the constrained model, where $\mu_{1},\mu_{2},$ and $\mu_{3}$ are the latent means for each group.

Next, to explore whether there were any differences depending on political affiliation, and any interaction effects, we estimated the full 3 × 2 × 3 between-subjects Bayesian ANOVA per outcome variable. For the ordinal models, we expected that the two-way interaction effects are half the size of the main effects, specifying a Cauchy prior such that 50% of the probability mass is within the [−0.15, 0.15] range. Similarly, we expected that the three-way interaction is half the size of the two-way interaction effects, specifying a Cauchy prior such that 50% of the probability mass is within [−0.075, 0.075]. This encoded the fact that interactions are generally smaller than main effects. Note that we did not specify the priors of the interaction effects in stage 1 of the registered report. The scaling by a factor of two strikes us as sensible but different choices are possible. As for the confirmatory analyses, we provided sensitivity analyses to assess the robustness of various prior widths (see the electronic supplementary material). The size of the interaction effects for donation behaviour, which we analysed using a Bayesian ANOVA via the R package *BayesFactor* [49], is not set directly but is specified hierarchically (for details, see [50]). We also provided sensitivity analyses for this outcome variable (see the electronic supplementary material). For donation behaviour, only the 3 × 2 × 3 model was run given that we did not formulate any specific hypotheses concerning the effects of the type of protest or scientist engagement. To adjust for multiple comparisons, we used Bayesian model-averaging, estimating a total of 19 models, ranging from a model with no main nor interaction effects to one that has all three main effects, all two-way and three-way interactions. We assigned a uniform prior to all models and followed the *principle of marginality* and did not include models that have an interaction effect without any of the constituent main effects [51,52]. We make model-averaged inferences by computing inclusion Bayes factors, that is, assessing the strength of evidence for a particular effect while taking into account the uncertainty across models [52,53]. Inclusion Bayes factors are defined as the ratio of posterior odds that a particular effect is present in the data compared with its prior odds. This approach solves the problem of what models to compare when assessing the evidence for a particular effect, taking all relevant models into account and thus leading to more robust inferences. Let ‘engagement’, ‘protest’ and ‘politics’ stand for the factors ‘type of scientist engagement’, ‘protest form’ and ‘political affiliation’. For example, to assess the evidence in favour of the effect of scientist engagement (‘engagement’), we do not only compare the model that includes ‘engagement’ to one that includes no effect at all (the null model); nor do we compare a model that includes ‘engagement + protest’ against a model that only includes ‘protest’. Instead, we are comparing all models that include ‘engagement’ with all models that do not include ‘engagement’, taking into account that the meaning of the effect ‘engagement’ changes when there is an interaction involving it by not including any such models in the comparison. To give a concrete example, the inclusion Bayes factor in favour of the effect of scientist engagement (‘engagement’) is computed as follows. First, we calculate the prior odds of ‘engagement’, that is, we divide the prior probability that ‘engagement’ is included in a model by the prior probability that it is not included in the model, while removing from the set of models those that include an interaction including ‘engagement’. The relevant set of models where ‘engagement’ is included is (‘engagement’, ‘engagement + protest’, ‘engagement + politics’, ‘engagement + protest + politics’, ‘engagement + protest + politics + protest: politics’), while the relevant set of models where ‘engagement’ is excluded is (null model, ‘protest’, ‘politics’, ‘protest+politics’, ‘protest+politics + protest:politics’). Since there are a total of 10 relevant models, each of which has the same prior probability, and there are 5 in the set of models that include and exclude ‘engagement’, respectively, the prior odds for the effect are 1. Second, we calculated the posterior odds of ‘engagement’, that is, we divided the posterior probability that ‘engagement’ is included in a model by the posterior probability that it is not included in the model, again removing any models from the set of models that include an interaction with ‘engagement’. To illustrate this, using a simulated data example with *n* = 150 and an effect size of δ = 0.20, we find posterior odds of 0.99997/0.000029 = 34 463. The inclusion Bayes factor is therefore 34 463/1 = 34 463.

When assessing the evidence for an interaction, say ‘protest : politics’, we had to be similarly careful which of the 19 models we include. The approach we followed is similar to the approach when assessing main effects: we compared models that include the interaction term with those that do not include it yet include all relevant main effects, that is, we again match the relevant models [52]. Calculating the inclusion Bayes factor for the effect ‘protest : politics’, we again have prior odds of 0.50/0.50 = 1 (as the relevant sets of models are (‘protest + politics + protest:politics’, ‘engagement + protest + politics + protest : politics’) and (‘protest + politics’, ‘engagement + protest + politics’)) and posterior odds of 0.0774/0.923 = 0.084, yielding an inclusion Bayes factor *against* the effect ‘protest : politics’ of 1/0.084 = 11.90.

**Figure 1.**
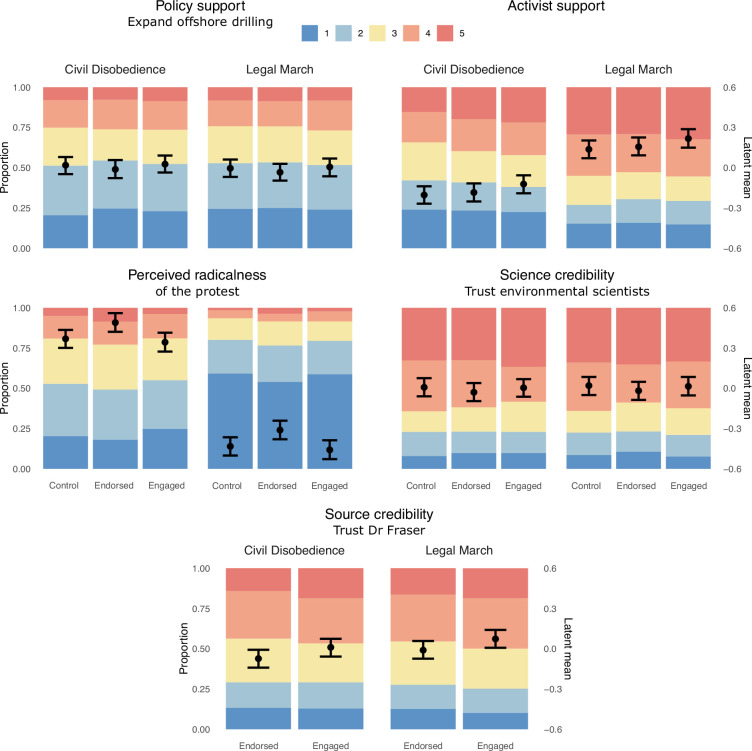
Likert responses and model-implied latent means for policy support (top left), activist support (top right), perceived radicalness of protest (middle left), science credibility (middle right) and source credibility (bottom). Likert response options for policy and activist support ranged from 1: strongly disagree to 5: strongly agree; for perceived radicalness from 1: not at all radical to 5: extremely radical; and for science and source credibility from 1: not at all to 5: a great deal.

**Figure 2.**
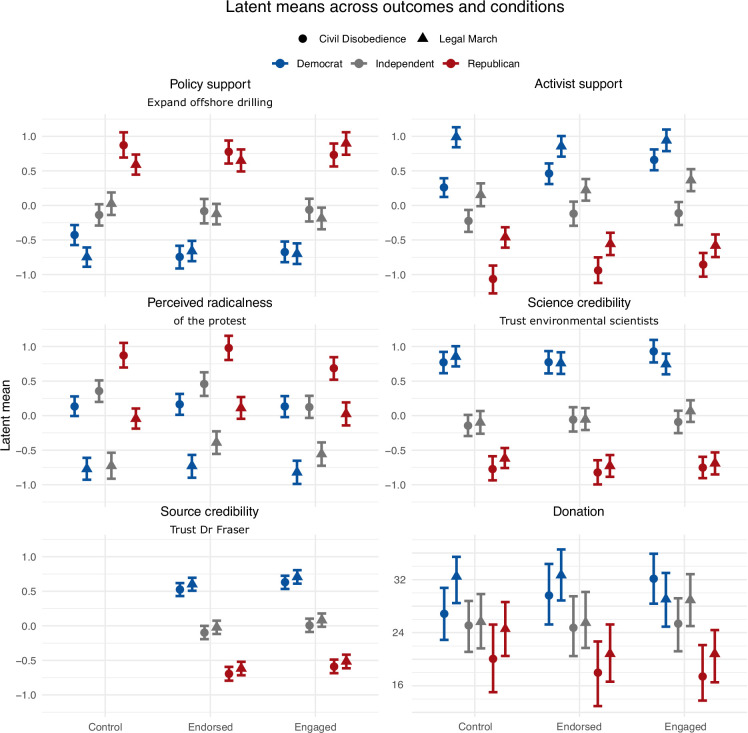
Latent means across political affiliation (blue: Democrat, grey: Independent, red: Republican), protest (circle: civil disobedience, triangle: legal march), and engagement form for policy support (top left), activist support (top right), perceived radicalness of the protest (middle left), science credibility (middle right), source credibility (bottom left) and donation behaviour (bottom right).

We used the R packages *brms* [43] to estimate Bayesian ordinal regression models and *bridgesampling* [54] to estimate Bayes factors. We used *marginaleffects* [55] to calculate model-implied latent means for the ordinal model that includes all main effects and interactions, visualized in figures 1 and 2. For donation behaviour, we used an ANOVA model with the same prior width [50]. We conducted a power analysis that informed our target sample size of *n* = 175 per condition, yielding *n* = 3150 in total. This corresponded to statistical power of almost 100% to find strong evidence in favour of an effect size as small as δ=0.20 in the two-group case and about 85% for the three-group case (with $\delta_{1}=\delta_{2}=0.10$ ). Statistical power to detect δ=0 was about 75% in the two-group case and almost 95% in the three-group case ( $\delta_{1}=\delta_{2}=0$ ); for details, see the electronic supplementary material.

### 2.3. Procedure

Participants were recruited via Prolific [56] between 25 and 28 October 2024. Prolific is an online research platform that allows for the recruitment of a representative US sample, based on age, sex and political affiliation.^1^ We aimed to recruit a total of 3150 US-American participants (see the electronic supplementary material, Sampling plan). We pre-specified a maximum recruitment period of two weeks, but recruitment was concluded within four days. Participants were rewarded an hourly wage of $12, resulting in a payment of $1 for a completion time of 5 min. After consenting to participate in the study, which was framed as being about views of the portrayal of climate protests in the media, participants were randomly assigned to one of the conditions and presented with the vignette text that described activists or activists and scientists engaging in a protest against offshore oil drilling (see the electronic supplementary material). Participants were told that they will be presented with a newspaper article, and asked to read it carefully as they would be asked to answer questions about it. To increase engagement with the vignette text, they were asked to briefly describe how the media portrayed the protest in 1−2 sentences in an open text box presented below the vignette text.

The items assessing the outcome variables (policy support, activist support, perceived radicalness of tactics and science credibility (general and source)) and the filler question (see below) were then presented in randomized order, except donation behaviour, which was always presented last. We assessed policy support by asking participants’ agreement for the item ‘offshore drilling for oil and natural gas off the US coast should be expanded’ on a five-point Likert scale (1: strongly disagree, 2: disagree, 3: neither disagree nor agree, 4: agree, 5: strongly agree). We chose this item because it has about half support and half opposition in a representative sample of the US population (https://climatecommunication.yale.edu/visualizations-data/ycom-us/). We assessed activist support via agreement to the five-point Likert item ‘How much do you support the activists described in the news article?’ (1: not at all, 2: a little bit, 3: a moderate amount, 4: quite a bit, 5: a great deal [30,35]). The perceived radicalness of the protest was assessed using an item based on Simpson *et al*. [57], asking ‘How radical do you think the protest described in the news article is?’ (1: not at all radical, 2: slightly radical, 3: moderately radical, 4: very radical, 5: extremely radical). To assess the credibility of the scientist engaging in action, we adapted an item from Friedman [31] and asked ‘How much do you trust Dr Fraser as a source of information about climate change?’ Note that this item was only presented in the scientist engagement conditions that include Dr Fraser, thus not in the control condition. To assess credibility in science more broadly, we adapted an item from [58] and asked ‘In general, how much do you trust environmental scientists as a source of information about climate change?’ Both credibility items were assessed on a scale from (1: not at all, 2: a little bit, 3: a moderate amount, 4: quite a bit, 5: a great deal). As a filler question, participants were asked ‘Do you find the portrayal of the climate protest in the news article you read balanced?’ (yes, no, don’t know). Finally, to assess donation behaviour, participants were told that a total of 10 participants who finish the survey will be randomly drawn to receive a bonus payment of $100 each. They could then choose to donate (a percentage of) the $100 bonus payment to one out of a number of not-for-profit organizations working on climate change (Sierra Club, Environmental Defense Fund, Rainforest Action Network, Friends of the Earth, Clean Air Task Force, 350.org, Center for Biological Diversity, Climate Emergency Fund), which they could indicate using a slider from 0−100. We distributed the bonus payment as decided upon by the selected participants, that is, the amount of money each participant decided to keep was paid out to them in the form of a bonus payment within Prolific, and the amount of money the participant decided to donate was donated to the chosen not-for-profit organization.^2^

Next, they were asked to provide information on the following demographics: gender (male, female, non-binary/third gender, prefer to self-describe, prefer not to say), age (<18, 18–24, 25 –34, 35–44, 45–54, 55–64, 65–74, 75+, prefer not to say), ethnic background (White, Black or African American, American Indian/Native American or Alaska Native, Asian, Native Hawaiian or other Pacific Islander, other, prefer not to say), gross annual income in dollars (less than 10.000, 10.000–19.999, 20.000–29.999, 30.000–39.999, 40.000–49.999, 50.000–59.999, 60.000–69.999, 70.000–79.999, 80.000–89.999, 90.000–99.999, 100.000−149.999, 150.000 or more, prefer not to say), education level (some high school or less, high school or General Educational Development (GED) diploma , some college but no degree, associates or technical degree, bachelor’s degree, graduate or professional degree (MA, MS, MBA, PhD, JD, MD, DDS etc.), prefer not to say) and political affiliation (Democrat, Democrat leaning, Independent, Republican leaning, Republican). Note that for the analyses below, we categorized participants who indicated that they lean Democrat as Democrats, and those that lean Republican as Republicans.

As an attention check, we asked participants the following question at the end of the survey: ‘What type of protest was described in the news article? (peaceful protest without arrests, blockade with arrests, there was no protest)’. Finally, we also asked the participants whether they had read about this protest before (given that it was a real protest). Upon finishing the full survey, participants were debriefed about the purpose of the study being about the effects of scientists engaging in different forms of climate protests on perceptions of climate change, activists and science credibility (see the electronic supplementary material). On the same page, they were provided with the opportunity to leave comments and feedback to the researchers in an open text box. All items of the survey were mandatory, meaning that participants could only finish the survey if they had answered all items.

### 2.4. Exclusion criteria

Participants were removed if they (i) did not finish the complete survey, (ii) provided an incoherent answer when describing how the media portrayed the protest described in the vignette text, and/or (iii) answered the attention check item incorrectly. For (ii), one coder (M.S.M.S.) determined whether answers were coherent (e.g. no gibberish answers, copying and pasting text from the survey, entering numbers, etc.; see [59]). A second coder (F.D.) then assessed any answers that the first coder indicated being unsure about, so as to jointly come to a decision as to whether to exclude that participant or not.

## 3. Results

### 3.1. Participants

A total of 3359 participants took part in the study (see the electronic supplementary material, Sampling plan). Participants were removed because they did not finish the survey (*n* = 210), they did not answer the attention check correctly (*n* = 274) or did not provide a coherent answer when describing how the media portrayed the protest described in the vignette text (*n =* 19; see Exclusion criteria and the electronic supplementary material). The remaining 2856 participants were included in the analysis. Out of this, 24% of participants were aged between 55 and 64 (*n* = 688), 50% were female (*n =* 1430), 73% described themselves as White (*n* = 2092), 12% reported having a gross annual income between $100.000 and 149.999 (*n* = 348), 36% have a bachelor’s degree (*n* = 1028) and 31% reported their political affiliation being Independent (*n* = 889). For a comparison of the sample demographics and recent US population estimates, see table 1, and the electronic supplementary material, table S2 for the complete sample demographics.

**Table 1.** Comparison of the sample and the US population.^3^

demographic variable	category	sample (%)	US population (%)
gender	female	50.1	50.5
	male	48.2	49.5
	other	1.6	NA
age^a^	18–24	11.7	11.6
	25–34	17.8	17.2
	35–44	17.1	17.1
	45–54	16	15.5
	55 and over	37.3	38.6
ethnicity	White	73.2	76.6
	Black or African American	12	13.4
	American Indian/Native American or Alaska Native	0.8	1.3
	Asian	6.3	5.8
	Native Hawaiian or other Pacific Islander	0.1	0.2
political affiliation^b^	Democrat	39.2	28.4
	Independent	31.1	42.2
	Republican	29.7	28.4
education level	some high school or less	0.6	NA
	high school diploma or GED	12.8	25.9
	some college, but no degree	21.3	18.9
	associates or technical degree	11.8	8.8
	bachelor’s degree	36	21.8
	graduate or professional degree (MA, MS, MBA, PhD, JD, MD, DDS, etc.)	17.3	14.3

^a^ Age categories reported here are based on the stratified age groups used in Prolific [61].

^b^ While we measured political affiliation on a five-point scale, we used the three-point scale for all analyses (see Methods) and report the recategorized variable here. See the electronic supplementary material for more information on the political affiliation categories. For the full demographics of the sample, see the electronic supplementary material, table S2.

### 3.2. Confirmatory results

Figure 1 shows the extent to which participants agreed with the policy to expand offshore oil and gas drilling (top left); to what extent they supported the activists who engaged in the protest (top right); how radical they viewed the protest (middle left); to what extent they trusted environmental scientists in general (middle right) and Dr Alex Fraser specifically (bottom) as a source of information. In addition to the descriptive statistics, the figure also shows the latent means across the conditions, using ordinal models that do not include political affiliation (see Exploratory results for analyses including political affiliation). The descriptives of all variables are reported in the electronic supplementary material, table S3.

Figure 1 suggests that policy support remained unchanged, while activists were more strongly supported when they engaged in a legal march than in civil disobedience. The latter was also rated much more radical than the former. The engagement of the environmental scientist did not seem to have an effect on either his trustworthiness nor on the trustworthiness of environmental science at large.

Bayes factor analyses confirm this visual impression and the mixed evidentiary situation for our confirmatory hypotheses (table 2). We found moderate evidence for protest form not having an effect on policy support (H1; Bayes factor BF_01_ = 8.06), and very strong evidence for the legal march drawing higher activist support (H2; BF_r0_ = 1.15 × 10^15^, BF_r1_ = 2) and for civil disobedience being perceived as more radical (H3; BF_r0_ = 3.31 × 10^78^, BF_r1_ = 2). However, we found very strong evidence against policy support being highest when scientists engage in the action followed by when they only endorse it followed by the control condition (H4; BF_0r_ = 43.95, BF_01_ = 38.90). We found moderate evidence against activist support being highest when scientists engage in the action followed by when they only endorse it followed by the control condition (H5; BF_0r_ = 4.30, BF_01_ = 14.43). We found very strong and weak evidence against the legal march and civil disobedience, respectively, being perceived as least radical when scientists engage in the action followed by when they only endorse it followed by the control condition (H6; BF_0r_ = 37.73, BF_01_ = 4.30 and H7; BF_0r_ = 1.78, BF_01_ = 0.69). Finally, we found weak evidence for the type of engagement having no effect on source credibility (H8; BF_01_ = 1.85) and very strong evidence of it having no effect on science credibility (H9; BF_01_ = 38.42). While the evidence against no effect on source credibility is weak, figure 1 suggests that, rather than having a negative effect on source credibility, engagement is more likely to have a positive effect. These conclusions are generally robust to different prior specifications (electronic supplementary material, figure S2).

**Table 2.** Evidence as quantified by the Bayes factor for our confirmatory hypotheses. (Bayes factors for H2 and H3 indicate comparisons of the constrained model against the null model. For those, the Bayes factor of the constrained model against the full model was 2, the theoretical maximum. Bayes factors for H4 to H6 show Bayes factors of the null model against the constrained model.)

hypothesis	Bayes factor	interpretation
H1: no effect of protest form on policy support	8.06	moderate confirmation
H2: higher activist support for legal march	1.15 × 10^15^	very strong confirmation
H3: higher perceived radicalness for civil disobedience	3.31 × 10^78^	very strong confirmation
H4: higher policy support for scientist engaging in, then endorsing, then control condition	0.023	very strong disconfirmation
H5: higher activist support for scientist engaging, then endorsing, then control condition	0.23	moderate disconfirmation
H6: legal march perceived as least radical when scientists engage in, then for endorsing, then for control condition	0.027	very strong disconfirmation
H7: civil disobedience perceived as least radical when scientists engage in, then for endorsing, then for control condition	0.56	weak disconfirmation
H8: no effect of type of engagement on source credibility	1.85	weak confirmation
H9: no effect of type of engagement on science credibility	38.42	very strong confirmation

### 3.3. Exploratory results

Next, we assessed whether our results vary across political affiliations and whether interaction effects exist by means of inclusion Bayes factors (see Analysis plan). We also provide inclusion Bayes factors for main effects, complementing our confirmatory analyses above. We found strong evidence for a main effect of political affiliation (table 3). Figure 2 shows the latent means across all conditions and outcome variables, indicating large effect sizes for political affiliation. Republicans showed the highest support for expanding oil and gas drilling, the lowest support for activists, viewed the protests as most radical, trusted Dr Alex Fraser and environmental scientists, in general, the least, and donated the lowest amount of money. Republicans are followed by Independents and then by Democrats in the expected direction.

**Table 3.** Evidence as quantified by the inclusion Bayes factor for all factors in our exploratory hypotheses. (Bold signifies evidence for the presence or absence of an effect with an inclusion Bayes factor larger than 10 (or smaller than 0.10), indicating (very) strong evidence.)

factor	policy support	activist support	perceived radicalness	source credibility	science credibility	donation
engagement	**0.04**	0.72	5.48	1.29	**0.04**	**0.09**
protest	0.37	**9.32 × 10^23^**	**1.81 × 10^89^**	0.43	0.22	4.73
politics	**3.65 × 10^158^**	**2.13 × 10^163^**	**1.18 × 10^47^**	**1.49 ×10^81^**	**1.60 × 10^183^**	**8.49 × 10^12^**
engagement × protest	0.31	2.11	0.35	0.24	**0.10**	**0.02**
engagement × politics	**0.06**	**0.03**	**0.04**	0.12	**0.03**	**0.004**
protest × politics	0.11	**0.09**	0.12	0.35	0.17	**0.01**
engagement × protest × politics	**10.16**	0.68	0.24	0.51	0.18	**0.02**

We found strong evidence against an effect of engagement on policy support (BF_incl_ = 0.04), science credibility (BF_incl_ = 0.04) and donation behaviour (BF_incl_ = 0.09), while the evidence for activist support, and source credibility was weak (BFs_incl_ of 0.72 and 1.29). We found moderate evidence of an effect of engagement on perceived radicalness (BF_incl_ = 5.48), but as the middle left panel in figure 2 shows, the pattern is not as we expected, with the endorse condition yielding the highest perceived radicalness. The overall highly similar pattern across engagement types for all panels in figure 2 indicates its small or non-existent effect. We found very strong evidence of protest type on activist support (BF_incl_ = 9.32 x 10^23^) and perceived radicalness (BF_incl_ = 1.81 × 10^89^), with large effect sizes as indicated by the latent mean differences between the civil disobedience (circle) and legal march (triangle) across engagement type and political affiliation (top-left and top-right panels in figure 2). The evidence for an effect of protest type on donation behaviour was smaller (BF_incl_ = 4.73), with its effect size indicating slightly higher donations for the legal march than the civil disobedience condition (bottom-right panel). Evidence against an effect of protest type on policy support (BF_incl_ = 0.37), source (BF_incl_ = 0.43) and science credibility (BF_incl_ = 0.22) was weak to moderate. There was strong evidence against the two-way interaction engagement × protest for science credibility (BF_incl_ = 0.10) and donation behaviour (BF_incl_ = 0.02), weak evidence against it for policy support (BF_incl_ = 0.31), perceived radicalness of the protest (BF_incl_ = 0.35) and source credibility (BF_incl_ = 0.24), and weak evidence in favour of it for activist support (BF_incl_ = 2.11). We found strong to very strong evidence against the two-way interaction engagement × politics for all outcome variables except source credibility, for which we found moderate evidence (BF_incl_ = 0.12). We found strong and very strong evidence against the two-way interaction protest × politics for activist support (BF_incl_ = 0.09) and donation behaviour (BF_incl_ = 0.01), respectively. The evidence against this interaction was moderate for policy support (BF_incl_ = 0.11), perceived radicalness of protest (BF_incl_ = 0.12) and science credibility (BF_incl_ = 0.17) and weak for source credibility (BF_incl_ = 0.35). We found weak to very strong evidence against all three-way interactions except for policy support, for which there was strong evidence in favour (see table 2). For Democrats and Republicans, policy support seems higher in the control condition for civil disobedience than the legal march, while the reverse is true for Independents. This pattern does not hold for the other two engagement types. These conclusions are generally robust to different prior specifications (electronic supplementary material, figure S3). The electronic supplementary material, figures S4–S8 show the raw proportions and latent means of the outcome measures across political affiliation, as in figure 1. The electronic supplementary material, figure S10 shows pairwise correlations between socio-demographic and outcome variables. Note that we had planned to use post hoc ordinal *t*-tests to assess group differences for effects with more than two levels. We decided not to do this for the two effects where this would have been relevant: the main effect of political affiliation, whose ordering is clear from figure 2, and the three-way interaction of political support, which would be difficult to interpret as we find no evidence of two-way interactions.

## 4. Discussion

A growing number of scientists have been following calls to ‘move out of the labs and into the streets’ and engage in climate protests, including civil disobedience, in order to emphasize the urgency of the climate crisis and to support the broader climate movement. However, the effects of such engagement on the general public’s climate change perception, behaviour and perception of science credibility remain critically understudied. In this study, we used a vignette approach in a large representative US sample (with respect to age and sex) to test whether protest type (legal march vs civil disobedience) and scientist engagement (control vs endorse vs engage) affect policy support, activist support, perceived protest radicalness, source and science credibility and donation behaviour.

In line with previous research, we found that protests are perceived differently by the general public depending on their type [30,35], with higher activist support and lower perceived radicalness for the legal march than civil disobedience. Yet, we found strong evidence that the protest type did not influence policy support for a policy directly related to the demands communicated by the protesters (i.e. to stop drilling for new oil and gas) and exhibited only a small effect on donations to a not-for-profit working on climate change. Our findings are in line with previous research showing that more radical tactics can lead to a decrease in support of the activists (e.g. [30]), but that there is no backfiring effect when climate groups use diverse tactics including civil disobedience [29,62], at least with respect to policy support.

Some have argued that scientists engaging in climate protests, in particular in acts of civil disobedience, may help communicate the urgency of the climate crisis and lend support to the wider climate movement (e.g. [12]). Our results do not speak to the former but do not support the latter, since we found evidence *against* an increase in stated policy support, donation behaviour, support of the activists and *against* a decrease in perceived radicalness of the protest when participants read about an environmental scientist endorsing or engaging in a protest. This is in contrast with earlier research showing that when an environmental scientist engaged in civil disobedience (versus in advocacy or teaching) there was an increase in participants’ risk perception regarding a phenomenon related to climate change [31].

Importantly, our results do suggest, however, that endorsing or engaging in protests does *not* lead to a backlash in the form of a decrease in science credibility. In line with studies investigating the effects of scientists advocating climate change [23–26,63] or engaging in civil disobedience [31], we found that scientists endorsing or engaging in climate protests do not erode science credibility. Similarly, while we found only weak evidence for our hypothesis that source credibility does not differ when a scientist endorses or actually engages in the protest, if anything our results suggest that it is higher when the scientist engages in the protest. Correspondingly, recent research has found that activism by scientists may even lead to higher science credibility in some countries while having no detrimental effect in any context [64]. The studies which did find that science advocacy can decrease science credibility highlighted important nuances concerning such negative effects. The effects depended on the solution advocated [27], the style of advocacy [28] and the topic at hand [23], indicating that such political engagement is not negative *per se*. As such, even providing information about the risks associated with politicized scientific issues can influence source credibility, the effect of which is moderated by political affiliation [65], but only on some topics (e.g. marijuana use), and notably not on climate risk information. Our findings suggest that the actions scientists can take in the face of the climate crisis, without having to fear a loss of credibility, are broader than just advocacy.

Not surprisingly, political affiliation emerged as the most important predictor for all outcomes, pointing to the importance of political polarization of perceptions around climate change, including policies, related protests and climate science, in the US context. Democrats emerged as most opposed to expanding offshore oil and gas drilling (cf [36,37,66,67]), most supportive of the activists [29,30] and showed the highest levels of science credibility [68,69]. Democrats were followed by Independents, with Republicans being most favourable to oil and gas expansion, least supportive of activists and most sceptical of science. We found generally strong evidence against the presence of interaction effects that include political affiliation. In other words, we did not find any indication of a potential backlash effect in the form of further polarization of such protests and/or scientist engagement, neither for Republicans nor for Independents.

Previous findings on interactions between political affiliation and scientist engagement are mixed. Panel data from shortly before and shortly after the ‘March for Science’ in 2017 showed a polarization of attitude towards scientists, but not on attitudes towards scientific research [38]. In other vignette studies on scientist engagement, some did find moderating effects of political affiliation [27,28], while others did not [23,26,31]. Similar to the potential negative effects of scientist engagement with political topics, such backlash effects may be dependent on the issue at hand as well as the style of engagement.

The results of the current study are based on a large US sample, which is representative with respect to age and sex and takes into account political affiliation, but the conclusions have to be interpreted with care given the following limitations. First, the manipulation of scientist engagement we used might not have worked, which could explain that it did not have an effect on any of the outcome variables. However, studies using similar vignette designs did find effects on comparable outcome variables (e.g. [23,26,28,29,31,65]). Additionally, in exploratory qualitative analysis of the open text response below the vignette (see the electronic supplementary material), we found that, across the scientist engagement conditions, 29.2% of participants mentioned a scientist engaging in or endorsing the action and 5.9% mentioned science in their description of the media’s portrayal of the protest, indicating that many participants did notice the explicit mentioning of the scientist when reading the short news article. The following could have reduced the effect of scientists’ engagement, however. All vignette texts included a short paragraph outlining some scientific background related to the protester’s demands, including what ‘scientists say’ about limiting warming and information from the International Energy Agency. This paragraph was added to increase the external validity of the vignette, since news articles often include scientific context when reporting on climate protests. However, 13.3% of participants in the control condition also alluded to science in their open answers. This may indicate that mentioning *actual* scientist engagement in addition to the scientific background related to the protest described may not have much of an additional effect on the outcomes we measured in this study. Future research that relies on vignettes may take these considerations into account in the study design.

We based our vignette texts on protests that occurred in New York City in the autumn of 2023, and aimed to make the differences between vignettes as minimal as possible. Future studies could employ more extensive material of scientist engagement to make it more salient, for example by using pictures or videos of a protest [30,35]. More in-depth knowledge about the potential effects of scientist engagement could also be gathered using interviews or focus groups on the topic [70]. Moreover, studies using survey panel data preceding and following protests that scientists publicly endorse and/or engage in may provide quantitative causal inference of the effects of such engagement (cf. [34,63,71,72]).

Second, while we based our power analysis on earlier findings of a vignette study of scientist engagement on climate risk perceptions [31], we found much smaller effect sizes. The evidence for (or against) the presence of effects of scientist engagement is therefore not conclusive for all outcome variables. One reason for the small effect sizes in this study may be that the outcome variables exhibited a higher skew than expected, limiting the variation available to detect an effect. For example, 53% of our sample (strongly) disagreed with expanding offshore oil and gas licensing, while only 26% (strongly) agreed with it. This is in contrast to the Yale climate opinion maps for 2023, which we based our item on and which showed that 56% supported expanding offshore oil and gas licensing while only 44% opposed it [73]. While more research with a more diverse sample (e.g. not recruited through Prolific but via nationally representative surveys) and potentially larger sample sizes is therefore warranted, the effects in question (as probed by vignette studies with all their limitations) may only be very small, if they exist at all. Third, the discrepancy with previous research may also be owing to different outcome measures. We chose to measure policy support, which strikes us as the most important variable. However, policy support has been found to be more difficult to influence than attitudes and beliefs [74]. Climate-related risk perceptions or a sense of urgency may be more malleable, which would explain the findings of Friedman [31], who found much larger effect sizes than present in our study. Future research may wish to measure both policy support and climate-related risk perceptions. Finally, we would like to note that our findings cannot confidently be generalized to other experimental stimuli (see [75,76]), including to other types of scientists (as types of scientists are evaluated differently by the general public, [77]).

Acknowledging the limitations of our study and taking our null results as true null results, our findings suggest that scientists can publicly engage in legal marches and (certain) acts of civil disobedience without having to fear that their actions may undermine their credibility and the credibility of science more broadly. The absence of a backlash on the credibility of both the scientist endorsing/engaging in the protest and the broader environmental science community of both Republicans and Independents is especially noteworthy, as arguments against the public engagement of scientists often allude to the potential of further polarization of trust in science and/or scientists particularly of this group (e.g. [21]). At the same time, scientists’ engagement in protests and civil disobedience may not be as impactful as previously suggested, as it did not increase opposition to offshore oil and gas drilling, activist support, donations and did not reduce perceived protest radicalness. This is in line with research on public health communication showing that while scientists may be seen as having more expertise, this may not translate into higher persuasiveness regarding the topic at hand [78].

## 5. Conclusion

Social change is hard—much harder than vignette studies like ours might inadvertently suggest. Will scientists joining protests or acts of civil disobedience galvanize the public into climate action? Evidently not—at least not by themselves. There are no shortcuts and no silver bullets. Tackling climate change requires the painstaking building of bottom-up power to, in the words of Bertrand Russell, ‘compel the so-called statesmen to acquiesce in measures that would make human survival possible’ [79]. This goes beyond mass protests and high-profile acts of civil disobedience. Our study suggests that scientists can engage in such actions without harming their credibility and the credibility of science more broadly. But it also suggests that scientists who do so should not overestimate their leverage; on its own, engaging in high-profile protests *as scientists* may have less impact on the public than one would like to believe.

## Data Availability

All data and code is available at [80]. Supplementary material is available online [81].

## Footnotes

^1^
While we aimed to recruit a representative sample including political affiliation, post hoc data quality checks showed inconsistencies concerning our measure of political affiliation and Prolific’s political affiliation categories. We therefore cannot be fully confident that our sample is representative in terms of political affiliation. See the electronic supplementary material for a discussion of this.

^2^
Based on the preferences of 10 randomly selected participants, a total of $310 (*M* = 31, s.d. = 22.82) was donated and the remaining $690 was paid out as bonus payments to the participants within Prolific. Of the donations, $80 was donated to the Environmental Defense Fund, $10 to Sierra Club, $90 to the Clean Air Task Force, $10 to the Rainforest Action Network and $120 to the Climate Emergency Fund.

^3^
US population estimates of gender, age, ethnicity and education level are based on US census data from 2021, see https://data.census.gov/table?t=-00. US population estimates of political affiliation are based on the average of Gallup data collected in 2024, see https://news.gallup.com/poll/15370/partyaffiliation.aspx [60].
